# Effects of Growth Hormone on Adult Human Gonads: Action on Reproduction and Sexual Function

**DOI:** 10.1155/2023/7492696

**Published:** 2023-04-07

**Authors:** Xin-Yi Zhou, Jia-Ni Ma, Ya-Yin Shen, Xue-Rui Xie, Wei Ren

**Affiliations:** Department of Endocrinology, The First Affiliated Hospital of Chongqing Medical University, Chongqing 400016, China

## Abstract

Growth hormone (GH), which is commonly considered to be a promoter of growth and development, has direct and indirect effects on adult gonads that influence reproduction and sexual function of humans and nonhumans. GH receptors are expressed in adult gonads in some species including humans. For males, GH can improve the sensitivity of gonadotropins, contribute to testicular steroidogenesis, influence spermatogenesis possibly, and regulate erectile function. For females, GH can modulate ovarian steroidogenesis and ovarian angiogenesis, promote the development of ovarian cells, enhance the metabolism and proliferation of endometrial cells, and ameliorate female sexual function. Insulin-like growth factor-1 (IGF-1) is the main mediator of GH. *In vivo*, a number of the physiological effects of GH are mediated by GH-induced hepatic IGF-1 and local IGF-1. In this review, we highlight the roles of GH and IGF-1 in adult human gonads, clarify potential mechanisms, and explore the efficacy and the risk of GH supplementation in associated deficiency and assisted reproductive technologies. Besides, the effects of excess GH on adult human gonads are discussed as well.

## 1. Introduction

Human anterior pituitary cells that can produce hormones include eosinophils (80%) and basophils (20%), and about 75% of eosinophils can secrete growth hormone (GH). Therefore, GH is the most abundant hormone present in the anterior pituitary gland of humans [1]. GH in humans is a 191-amino acid protein, with a molecular weight of 22 KD, which binds to the growth hormone receptor (GHR), and the structure of GHR is similar to that of the prolactin receptor [2, 3]. Insulin-like growth factor-1 (IGF-1), a single-chain polypeptide with a molecular weight of 7.7 KD in humans, is produced by target cells, typically hepatic cells, which are induced by GH [2, 4, 5]. GH and IGF-1 play roles in promoting growth and metabolism which contribute to hyperglycemia, lipolysis, and protein anabolism. In addition, they have important effects on adult gonads through direct and indirect manners. In this review, we shed light on the effects of GH and IGF-1 on reproduction and sexual function of adult humans and analyze the efficacy and risk of GH in the treatment of related deficiency and assisted reproductive technologies. Moreover, we also consider the situation of excessive GH.

## 2. Main Text

### 2.1. Puberty

The interaction of the hypothalamic-pituitary-gonadal (HPG) and hypothalamic-pituitary-somatotropic (HPS) axes in puberty contributes to the understanding of the effects of GH and IGF-1 on adult human gonads. It is reported that GH is a significant promoter of the onset of puberty and has vital effects on sexual maturation in all mammalian species [6]. The activation of the gonadotropin-releasing hormone (GnRH) pulse generator at the pubertal onset, which triggers the hormonal cascade essential for sexual maturation, is related to the body mass and nutritional state. Therefore, anabolic hormones such as insulin and IGF-1 may be responsible for contributing to GnRH pulsatility [7]. However, there are opposite results. Studies in GH-deficient and GH-resistant children demonstrate that IGF-1 may not normalize the time of pubertal onset [8]. A study has shown that only when the puberty pattern of pituitary gonadotropin secretion is established, GH can promote puberty development [9], and it may be related to the changes in follicle-stimulating hormone (FSH) secretion and luteinizing hormone (LH) action [10] or gonadal steroidogenesis with altered GH action [11]. The interaction between these two axes is complex, and it is still unclear which axis acts earlier and which axis dominates.

Delayed puberty is found in humans with Laron dwarfism who are insensitive to GH [12, 13] due to a mutation in GHR [14] or STAT5b (a signal transducer and activator of transcription 5b) [15] genes. For males, studies indicated that appropriate testicular function development in Laron dwarfism and GHR knockout mice was later than that in normal males but still occurred [8]. For females, studies in animals showed that functional GHR absence caused delayed pubertal onset and a decreased number of ovarian follicles, but they still had the ability to reproduce [16–19]. In humans with Laron syndrome, both male and female patients have not shown a particular decline in the reproductive capacity, though they have delayed puberty [19, 20]. Evidence from animals and humans with Laron syndrome suggests that GH may not be an indispensable factor for reproduction, but it is able to influence gonadal development. However, Laron syndrome is merely one of the conditions to evaluate the effects of GH on the gonads. The effects of GH on adult human gonads are elaborated below.

### 2.2. Effects of GH on the Gonads of Adult Males

#### 2.2.1. Reproduction of Males

GH and IGF-1 from different sources play roles in the testis in different ways including endocrine, paracrine, and autocrine ways [6]. The specific structure of the testis includes the seminiferous tubules and the surrounding connective tissue. The seminiferous tubules are mainly composed of peritubular myoid (PTM) cells, Sertoli cells, and germ cells. Leydig cells, blood vessels, lymph vessels, and nerves are located in the interstitial space between the seminiferous tubules. PTM cells surround the outer wall of the seminiferous tubule and cooperate with Sertoli cells to contribute to the basement membrane of the seminiferous tubule which is between the PTM and Sertoli cells. Sertoli cells surround developing germ cells and protect these cells [21, 22]. The blood-testis barrier (BTB) in the mammalian testes is formed by specialized junctions between adjacent Sertoli cells near the basement membrane of the seminiferous tubule. It divides the seminiferous epithelium into the basal and adluminal compartments. Preleptotene spermatocytes are the germ cells in transit at the BTB, from the basal compartment to the adluminal compartment, which will continue the process of differentiation into zygotene and diplotene spermatocytes, two meiotic divisions, spermiogenesis, and spermiation in the protected microenvironment [23]. The BTB restricts substances (e.g., nutrients, hormones, and electrolytes) into the adluminal compartment, so Sertoli cells are responsible for the supply of nutrients and the production of signaling molecules, which are essential for the process of spermatogenesis. However, in the testes of the primates, the myoid cell layer has a poor effect in restricting the passage of electron-dense substances through the seminiferous tubule [24], and it may be the same in human testes. In addition, the endothelial tight junction barrier in the microvessels has little contribution to the BTB function in the primates [25, 26]. Therefore, nutrients and hormones from the microvessels are not restricted to spermatogonial renewal, mitotic proliferation, and differentiation which take place in the basal compartment [27].

**Figure 1:**
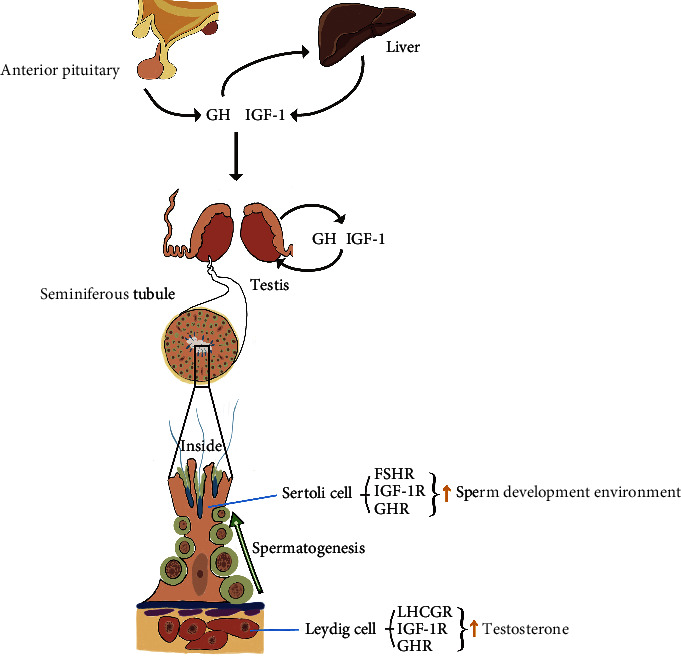
GH/IGF-1 roles in reproduction of adult males and potential mechanisms. GH secreted by the anterior pituitary, which is the main source of systemic GH, induces the liver to produce IGF-1. Systemic GH/IGF-1 reaches the testis and improves the production of local GH/IGF-1. In response to systemic GH/IGF-1 and/or local GH/IGF-1, FSH action on the Sertoli cell is improved to ameliorate the sperm development environment, and LH action on the Leydig cell is promoted to increase the levels of T. The effect of GH/IGF-1 on spermatogenesis is not clear and is not shown in the figure. GHR: growth hormone receptor; IGF-1R: insulin-like growth factor-1 receptor; LHCGR: luteinizing hormone/choriogonadotropin receptor; FSHR: follicle-stimulating hormone receptor.

As described above, the myoid cell layer and the endothelial tight junction barrier have few effects on the restriction of the substance passage. Accordingly, circulating GH and IGF-1 (mainly incorporating pituitary GH and GH-induced hepatic IGF-1) released from the blood vessels can reach the Leydig cells in the interstitial space and cross the myoid cell layer to Sertoli cells and the basal compartment. The GHR and IGF-1 receptor (IGF-1R) are observed to be expressed in both Leydig cells and Sertoli cells [28–30] (Figure 1), so that circulating GH and IGF-1 can act on the Leydig cells and Sertoli cells directly. Circulating GH and IGF-1 may affect the development and differentiation of spermatogonia in the basal compartment [28, 30]. The myoid cell layer and the endothelial tight junction barrier have little restriction in circulating GH and IGF-1. As a consequence, the ability of circulating GH and IGF-1 to cross them may be similar [31], but circulating GH and IGF-1 cannot penetrate the BTB. GHR and IGF-1R are also found in germ cells [28–30], so there appears to be a strong possibility that GH and IGF-1 are produced by Sertoli cells and act on the germ cells through paracrine manners [32] even act upon themselves via autocrine manners. Moreover, systemic GH and IGF-1 may have indirect impacts on germ cells by affecting the anabolism of Sertoli cells.

There is evidence that GH gene expression has been found within the human testis [33, 34]. There are two clinical versions of GH, containing the normal GH form (GH-N) and the variant GH form (GH-V). GH-N is secreted from the pituitary gland and has been detected in the human testis [34]. The products of the GH-V gene, which were thought to be pregnancy-specific previously [11], are the most abundant GH messenger RNA (mRNA) isoform in the human testis [35]. Cellular localization of GH in the human testis is unclear [8], but the “trace” of GH regulators is found in specific cells. The expression of growth hormone-releasing hormone (GHRH) receptors is observed in Leydig cells, Sertoli cells, and spermatogonia of humans [36]. GHRH immunoreactivity and GHRH mRNA are detected in the human testis [36, 37], and testicular GHRH is able to stimulate GH synthesis [6]. GHRH may act via its receptor in the testis exerting direct effects on testicular cells and/or playing indirect roles by promoting the production of testicular GH. Ghrelin is the endogenous ligand for the GH secretagogue-receptor (GHS-R), and GHS-R type 1a (GHS-R1a) is the functionally active form of GHS-R [38–40]. In the human testis, ghrelin immunoreactivity is located in Leydig cells and Sertoli cells, and the GHS-R1a protein is detectable in germ cells, Leydig cells, and Sertoli cells [41].

With respect to the capacity of testicular cells to synthesize GH, it is inferred based on the data obtained from the animal model. One study explored the effects of human chorionic gonadotropin (hCG, as an agonist of LH) and FSH on regulating the expression of GHS-R1a, respectively, in the rat testis. The result showed that FSH improved GHS-R1a mRNA expression significantly, whereas hCG played no role in modulating the GHS-R1a transcript [42]. Therefore, FSH may stimulate the expression of GHS-R1a in Sertoli cells, since the FSH receptor (FSHR) is located in Sertoli cells [6]. LH and hCG bind to a common receptor, the LH/choriogonadotropin receptor (LHCGR) [43, 44]. The distribution of the LHCGR is in Leydig cells [6], so that hCG is not able to change the levels of GHS-R1a in Leydig cells. In this regard, it is speculated that Sertoli cells may be more capable of producing GH than Leydig cells. It is worth noting that the possible cellular localization of GH in the human testis and the GH synthesis ability of testicular cells are analyzed and speculated based on potential regulatory factors which may stimulate testicular GH production. Although the direct action of gonadotropins on corresponding target cells is considered, indirect effects on other cells may also be involved. Hence, immunohistochemical methods and in situ hybridization are required to confirm the cellular distribution of GH in the human testis, and the potential mechanism of hormonal interactions remains to be explored.

IGF-1 has been detected in Sertoli cells, Leydig cells, and primary spermatocytes by immunohistochemistry in humans [30, 45]. In the testis, gonadotropins mainly regulate the synthesis of IGF-1, and GH also has effects on local IGF-1 production albeit the effects are not as significant as gonadotropins [46]. *In vivo*, it could not be determined which gonadotropin (FSH or LH) played a more apparent role in testicular IGF-1 synthesis, since the experimental results were inconsistent [47, 48]. *In vitro*, FSH increased IGF-1 secreted by rat Sertoli cells, and IGF-1 produced by rat Leydig cells was improved through the stimulation of LH. In the cultures where Sertoli cells and Leydig cells coexisted, the production of IGF-1 exceeded the expectations from monocultures both under the basal conditions and after the stimulation of gonadotropins, suggesting that the interaction of the Sertoli cell and the Leydig cell may be prominent for the production of testicular IGF-1 [49]. IGF-1 plays important roles in the testis. Men with distal chromosome 15 structural abnormalities have a higher probability of experiencing low testicular volume, cryptorchidism, and oligoasthenoteratozoospermia. This may be a result of the deletion of the IGF1R gene locus [50]. A study enrolled seven males with Laron syndrome who were treated with IGF-1, showing an increase in testicular volume, gonadotropin levels, and testosterone (T) levels [51]. It has been reported recently that IGF-1 supplementation increased the sperm concentration, sperm volume, and sperm progressive motility of an infertile man [52]. Moreover, it was also found *in vitro* that IGF-1 could augment the progressive motility and vitality of human spermatozoa which were positively correlated with sperm fertilizing capacity [53]. However, a study *in vitro* showed the discordant data. It was reported that IGF-1 significantly decreased several motility parameters of human spermatozoa including the curvilinear velocity and the amplitude of lateral head movement [54].

Interconnection of the HPG and HPS axes is significant in adulthood as well. On the one hand, GH and IGF-1 are able to play roles on different levels of the HPG axis including action on GnRH neurons, gonadotrophic cells, and testes. Therefore, GH and IGF-1 can modulate steroidogenesis and may influence spermatogenesis [55]. Idiopathic/isolated hypogonadotropic hypogonadism (IHH) is caused by impairment of GnRH neuronal development and migration, GnRH secretion, or GnRH action [56]. A study in 2021 has shown that GH and IGF-1 are able to support the migration and secretory function of both immature and mature GnRH neurons [57]. Moreover, a study suggests that a mutation of the IGF1 gene may lead to IHH [58]. Consequently, IHH and GH deficiency (GHD) may be closely related. In clinical practice, when a person is diagnosed with IHH, it is necessary to be tested for GHD. GH supplementation may be a therapeutic choice for patients with IHH. On the other hand, T and estradiol (E2) through aromatization from T can promote the secretion of GH. T induces GH secretion via regulating ghrelin which can stimulate GH release [59]. In males, E2 can increase the levels of GH through the estrogen receptors after aromatization from T [60]. A study has found a direct effect of E2 on GH synthesis in somatotrophic cells via *α* and *β* estrogen receptors [61]. Men with an absence of E2 due to aromatase deficiency appeared to have impaired GH secretory reserve which was in line with GHD. Even though IGF-1 levels were within the normal range, they were markedly lower than those in normal men. The situations remained unchanged during E2 supplementary treatment, indicating that E2 has beneficial actions in the development and maturation of the GH-IGF-1 axis [62, 63]. The interaction of the HPG and HPS axes suggests that there may be a close connection between GHD and hypogonadotropic hypogonadism (HH) in adult males.

GH and IGF-1 have been reported to increase gonadotropin sensitivity. Gonadotropins secreted by the anterior pituitary mainly contain LH and FSH. Low levels of hCG (probably from the pituitary) are found in men [64]. Although hCG is not the key driving force of endogenous steroid production, exogenous hCG can be used to improve T production in cases of male hypogonadism and infertility [65]. The LHCGR is expressed in Leydig cells, and the FSHR is detected in Sertoli cells. GHR is localized in both kinds of cells. In Leydig cells, GH acts in synergy with LH to increase T synthesis and secretion. Furthermore, GH has independent effects on steroidogenesis. GH enhances the expression of the steroidogenic acute regulatory protein (StAR, a kind of precursor substrate for sex steroid synthesis) and 3*β*-hydroxysteroid dehydrogenase (3*β*-HSD, a kind of steroidogenic enzyme) in Leydig cells [11, 66, 67]. In Sertoli cells, GH cooperates with FSH to improve the sperm development environment, promote the early development of spermatogonia, and ensure their complete maturation [68] (Figure 1). The IGF-1R is expressed in Leydig cells and Sertoli cells [29, 30], and IGF-1 is also responsible for interaction with gonadotropins [5, 49] (Figure 1).

In terms of GH roles in T synthesis, data from clinical studies are heterogeneous. In males with isolated GHD, a study showed that hCG-stimulated T levels rose markedly after recombinant human growth hormone (rhGH) treatment when compared to the study entry, suggesting that rhGH treatment could increase testicular steroidogenesis by improving the response of Leydig cells to hCG [69]. In a study, patients with both GHD and deficiency of gonadotropins were under gonadotropic treatment and GH therapy was added for a period of time. The result showed that T production of these patients increased more significantly during GH administration [65]. T secretion of men with isolated HH who had a poor response to gonadotropins increased after the combined treatment of gonadotropins and GH [70]. Nevertheless, a study indicated that T levels of GHD patients remained unaltered after GH treatment [71]. Another study enrolled men with oligozoospermia but without GHD indicated that short-term GH cotreatment with hCG almost had no effects on seminal IGF-1 concentration and steroidogenesis compared to utilizing hCG alone [72]. In addition, the degrees of total serum T and serum sex hormone-binding globulin (SHBG) declined after GH treatment, which was given to middle-aged overweight men [73]. Another study demonstrated the same results, in which adult males with organic GHD but otherwise normal HPG axis were enrolled, but calculated free T (cFT) did not change [74]. It is suggested that the evaluation of the HPG axis via cFT rather than total T is essential to determine whether GH treatment is effective or not, since some studies have shown that GH may enhance the action of T by reducing the production of SHBG which decreases the bioavailability of T [8, 75]. Indeed, GH treatment may not increase the T levels in patients with GHD and/or HH in some cases. Some factors should be taken into account, such as the duration of GH administration and the levels of SHBG. It offers a choice that examinations about GH deficit should be considered and that GH treatment may be given when patients with HH are resistant to gonadotropin therapy.

GH may be involved in spermatogenesis and may have positive effects on sperm parameters. In animal models, studies demonstrated that GH could improve spermatogenesis [76, 77]. It has been reported that the possible mechanism by which GH may enhance spermatogenesis is through the stimulation of Leydig and Sertoli cell differentiation [6]. However, the potential benefits of GH on spermatogenesis in human subjects have not been shown in large randomized trials. Besides, data on the impacts of GH therapy on spermatogenesis are controversial. GH1 gene mutations, especially GH1 gene defects, can cause severe GHD [78, 79]. Many cases were first seen in childhood with short stature, and reports about adult cases are scarce. GH treatment may be of use at the beginning, but it becomes ineffective subsequently since GH protein is immunogenic and GH antibodies are produced [79]. One case of a patient, with a homozygous 6.7 kb GH1 deletion, who was treated with recombinant human IGF-1 (rhIGF-1) at the age of 16.7 years after the failure of GH treatment, attained a perceptible growth response and had increased testicular volume (from 4 ml to 10 ml). Subsequently, the clinical benefits of IGF-1 therapy were not evident, and the testicular volume remained 10 ml [79]. Another case was a 56-year-old male with a GH1 deletion. He accepted GH treatment when he was 7 years, and GH antibodies were detected as well; thus, the treatment was discontinued. However, the male had two sons and did not have the clinical expression of hypogonadism. The testicular volume of the man was 20 ml [80]. Data on sperm parameters in these patients have not been mentioned in the related studies. Therefore, it is postulated that the testicular volume of some patients with GHD caused by GH1 gene defects may be lower than that of normal males and that they may have lower sperm concentration and lower percentage of progressive motility and spermatozoa with normal form than males with normal testicular volume, indicating that the spermatogenesis capacity of these patients may decline [81, 82]. Nevertheless, some patients may have no sign of a decrease in reproductive ability [80, 83]. For these cases, continuous follow-up and deep exploration are needed and sperm parameters and fertility in adulthood are supposed to be evaluated.

Some studies have provided pieces of evidence that rhGH therapy can improve sperm concentration, total sperm count, sperm motility, and sperm survival rate in patients with idiopathic oligozoospermia or idiopathic asthenospermia and obese men with oligoasthenospermia [84–86]. Congenital combined pituitary hormone deficiency (CCPHD) is a relatively ideal model to investigate the condition when GHD and HH coexist, because almost 100% of patients with CCPHD have GHD, and HH happens in 96% of patients with CCPHD. Additionally, complete hormonal deficiency happens when individuals with CCPHD are born. During adulthood, patients with CCPHD who accepted gonadotropins combined with rhGH achieved earlier spermatogenesis and had a higher spermatogenesis rate than patients who only accepted gonadotropin therapy. Notably, the study has also confirmed that rhGH treatment is an independent factor which is conducive to stimulating spermatogenesis [87].

Several studies have demonstrated that GH can affect some sperm parameters. The effects of GH treatment on spermatogenesis in subfertile men with relative GH insufficiency and FSH elevation were investigated in a study. The subfertile men were divided into the two groups: subjects with oligozoospermia and subjects with asthenospermia. Asthenospermic subjects attained more significant increased sperm motility, and the elevation of semen volume was more significant in the oligozoospermic group, while the alteration of the sperm count was not found in these two groups. Still, in this study, 33% (3/9) of patients in the asthenospermic group made their wives pregnant and healthy children were born, whereas there were no pregnancies in the oligozoospermic group [88]. One study that involved men with severe idiopathic oligozoospermia who had normal gonadotropin levels and a normal GH response showed that, despite no increase in the sperm count or semen volume under GH treatment, sperm velocity was improved during the GH therapy [89]. Other studies found no influence of GH on spermatogenesis. A study mentioned above also showed that rhGH treatment did not increase the sperm count and motility of males diagnosed with isolated GHD [69]. One study, in which 4 hypogonadotropic, hypogonadal men who had azoospermia accepted gonadotropin therapy for 6 months and accepted cotherapy of GH and gonadotropins for the next 6 months, demonstrated that all patients remained azoospermic [90]. In addition, GH treatment had no effect on spermatogenesis in normogonadotropic men with severe oligoteratoasthenospermia [91]. From the results of GH treatment, the positive effect of GH supplementation on sperm motility is relatively prominent. However, the small size and different treatment protocols of these studies preclude the ability to make recommendations regarding the use of GH in the treatment of male fertility. Moreover, it cannot be concluded for whom GH treatment on spermatogenesis is effective and for whom it is ineffective.

Data on reproduction of men with excess GH are paradoxical. A study published in 2002 analyzed 35 acromegalic patients, which showed lower levels of the seminal volume, sperm counts, and total motility in acromegalic men than those in controls, and reduced concentrations of T were found in acromegalic men. After GH/IGF-1 suppression treatment, sperm counts, total motility (in patients who achieved disease control), and T levels increased [92]. The results implied that excess GH had negative effects on spermatogenesis and T production and that the effects of lowering GH and IGF-1 in acromegalic men were obviously positive. In contrast, a recent study demonstrated that there were no differences in regard to seminal volume, sperm counts, or total motility between acromegalic men and healthy men, albeit acromegalic men presented lower levels of serum T and cFT [93]. Further studies are supposed to focus on the reproduction of acromegalic patients.

#### 2.2.2. Sexual Function of Males

The sexual function of males can be assessed by the validated International Index for Erectile Function-15 (IIEF-15) which is a self-administered questionnaire containing 15 questions related to erectile function (EF), sexual desire, orgasmic function, intercourse satisfaction, and overall satisfaction [94, 95]. Higher scores indicate better sexual function. A recent study demonstrated that the prevalence of erectile dysfunction (ED) in 52 male patients with adult GHD (AGHD) was 60% according to the EF score of the IIEF-15 questionnaire. ED is particularly prominent in AGHD patients with sexual dysfunction. A significantly lower ED prevalence was found in rhGH-treated males (35%) than that in untreated males (75%) [96]. The study also suggested that aging and low-level T were less correlated to worse IIEF-15 scores than AGHD and low-level IGF-1 [96].

Penile erection is a result of neural, psychological, and hormonal regulation. Sexual stimulation causes the release of nitric oxide (NO) from the nonadrenergic and noncholinergic (NANC) nerves in penile tissue, which is enhanced by GH and T. Nitric oxide (NO) combines with soluble guanylate cyclase to increase 3′, 5′-cyclic guanosine monophosphate (cGMP), which activates protein kinase G (PKG) to form a complex cGMP/PKG and reduces intracellular calcium levels, promoting the relaxation of the cavernous smooth muscle, expanding the cavernous arteries, and leading to penile erection [97]. ED refers to the inability to continuously obtain or maintain penile erection sufficient for satisfactory sexual performance [98]. The disorder of GH secretion could lead to the impairment of erectile function, since the serum GH levels in the cavernous and systemic blood cavities of men with organic ED were lower than those in healthy men during sexual arousal with penile tumescence [99]. A study investigated the NO levels in patients with AGHD, suggesting that NO formation increased in rhGH-treated men, while it decreased in untreated men who may have a higher risk of experiencing ED [100]. GH might have a direct influence on promoting the expression and activity of endothelial NO synthase (eNOS) in the vascular endothelium. Subsequently, the guanylate cyclase activity, which relies on NO, is stimulated in the vascular and nonvascular smooth muscle of the penile corpus cavernosum [101, 102]. Recent evidence suggests that GH plays an active role in maintaining the dynamic balance of the vascular endothelium, increasing the production of NO, and protecting the endothelium by regulating oxidative stress [103].

Furthermore, in men aged 60 or older, the decline in physiological GH associated with aging can be considered a possible cause of aging-related decreased sexual desire, decreased frequency of erection and sexual intercourse, difficulty in orgasm, and impaired sexual satisfaction [104]. In a study, 15 middle-aged and elderly men (42 to 77 years) were treated with a placebo in the first month and low-dose GH in the second and third months. The results showed that the sexual function score of patients treated with GH increased significantly in the two months, indicating that low-dose GH supplementation could improve the sexual function of some middle-aged and elderly men [105]. Nevertheless, a small controlled trial found that GH administration had no effect on the sexual function of healthy older men [106]. In general, trials with a larger sample size are needed to strengthen the promotional effect of GH on the sexual function of adult males.

Decreased sexual desire and ED are rather frequent in men with excess GH as well [107]. A study reported 62.7% (32/51) of patients with acromegaly had ED [108]. Compared with ED patients without acromegaly, patients with excess GH had a higher organic component of ED according to the Structured Interview on Erectile Dysfunction (SIEDY) [109], which was used to evaluate ED-related morbidities and emphasized the important role of organic components in the pathogenesis of dysfunction [110, 111]. This may be a result of endothelial dysfunction caused by metabolic complications of acromegaly and/or the direct impaired effect of excessive GH [112]. A study showed the severity of ED in acromegalic patients was related to GH levels, and acromegalic patients had significantly lower NO levels than nonacromegalic controls, supporting that excessive GH may contribute to endothelial dysfunction [108].

The indirect roles of GH disorders in sexual dysfunction should be considered. Several symptoms caused by AGHD, including physical decline, depression, and fatigue, are able to harm sexual function [113]. Sexual dysfunction in acromegalic patients can be attributed to the dissatisfaction of body image [114]. Furthermore, both GHD and acromegaly are able to lead to the decline of T levels. T is involved in regulating penile vascular endothelial function [115], and ED is more common in men with lower T levels [116]. Hyperprolactinemia is closely related to hypoactive sexual desire [117] and may cause ED. Due to the similarity of GH and prolactin receptors, excessive GH may mimic the role of prolactin [3] to induce decreased sexual desire and ED [107, 118]. It is controversial that whether excessive GH has direct effects on sexual function or other hormonal disorders play roles. The association between GH and sexual function is encouraged to be explored deeply.

### 2.3. Effects of GH on the Gonads of Adult Females

#### 2.3.1. Reproduction of Females

GH secreted by the anterior pituitary gland is the main source of circulating GH. It promotes the expression of hepatic IGF-1 and ovarian IGF-1. Furthermore, ovarian IGF-1 can also be also induced by FSH, LH, and E2 [46, 119]. GH and IGF-1 can stimulate uterine and ovarian function, since the expression of the GHR and IGF-1R is found in these tissues [120]. GH and GHR are present in pregnant and nonpregnant uteruses, and GHR, which is detectable throughout the uterine layers of women, is differentially regulated during pregnancy and the menstrual cycle [121]. GH can promote uterine growth, since the uteruses of GH-deficient women are smaller than those of GH-replete women under the condition of the corrected body surface area [122]. Low levels of GH gene transcripts and proteins were found in the human ovary, suggesting that GH may be produced in the ovary [123]. However, other data about GH of ovarian origin are so limited that the conclusion is not fully confirmed and that further studies are required. GHR is detected in various cells in the ovary [124, 125]. In addition, a paper published in 2019 indicates that the interaction of GH and local-produced ovarian factors including vascular endothelial growth factor-A (VEGF-A), fibroblast growth factor-2 (FGF-2), and IGF-1 contributes to ovarian angiogenesis [126].

**Figure 2:**
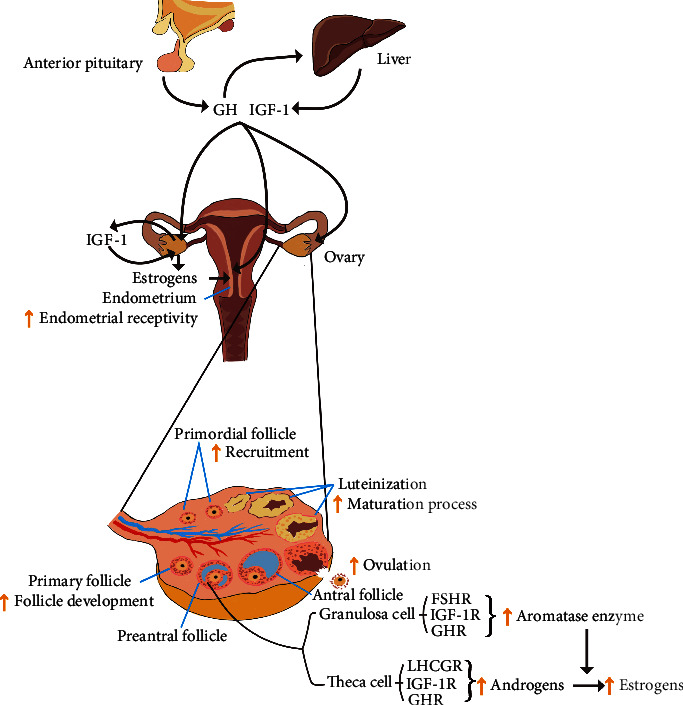
GH/IGF-1 roles in reproduction of adult females and potential mechanisms. Systemic GH/IGF-1 stimulates ovarian IGF-1 production. GH of ovarian origin is not fully confirmed and is not shown in the figure. Systemic GH/IGF-1 and/or ovarian IGF-1 play roles. They contribute to the recruitment of primordial follicles and promote the growth and development of follicles. They are able to improve the FSH action on the granulosa cell and promote the LH action on the theca cell. Subsequently, steroidogenesis is ameliorated. They can also induce ovulation and support the maturation process of luteinization. The effect of GH/IGF-1 on the endometrium is to improve endometrial receptivity. GH/IGF-1 can also contribute to an increase in endometrial receptivity by raising estrogen levels. GHR: growth hormone receptor; IGF-1R: insulin-like growth factor-1 receptor; LHCGR: luteinizing hormone/choriogonadotropin receptor; FSHR: follicle-stimulating hormone receptor.

GH can modulate the synthesis and release of sex steroids in follicles. In animal models, studies have shown that GH can contribute to the expansion of granulosa cells and theca cells, but it is less clear whether the effect is direct or indirect [127, 128]. GHR is expressed in granulosa and theca cells [124, 125]. GH is able to improve the role of FSH and the density of LHCGR in granulosa cells and promote the LH action on theca cells [129–131]. Androgens are produced by theca cells after LH stimulation, which are transformed into various estrogens through the aromatase enzyme expressed in granulosa cells via FSH induction [132]. Before oocyte release, granulosa cells become luteinized by increasing LHCGR density and decreasing preovulatory FSHR expression, which may be affected by local IGF through paracrine action when promoting the expansion of granulosa cells. The process is also regulated by hCG and granulosa lutein cells which are differentiated from granulosa cells produce progesterone [128, 130, 133]. Both *in vivo* and *in vitro* studies in women with reduced ovarian reserve have shown that GH supplementation in *in vitro* fertilization (IVF) treatment can increase the expression of the LHCGR, FSHR, and GHR in human granulosa cells [130, 134] (Figure 2).

GH is able to promote follicular development and improve oocyte quality. Studies in animals have shown that GH can contribute to the growth and development of the primordial follicles, and consequently, it is possible that it regulates the recruitment of the primordial follicles into the growing, gonadotropin-sensitive pool [135, 136]. GH also plays a role in follicular selection, since GH-binding sites in granulosa cells are lost in the atretic follicles [137]. In humans, a study has shown that GHR is present in female adult oocytes, granulosa cells, and stromal cells [125]. It is plausible to assume that GH may influence the development of follicles of adult humans indirectly and directly. The quality of oocytes harvested from follicles containing more GH in follicular fluid is higher, and a rise in the GH concentration at the early stage of small antral follicles is conducive to the quality of oocytes, by enhancing or cooperating with gonadotrophin-controlled developmental processes. In fertilized oocytes, the concentration of GH in follicles is negatively correlated with subsequent cleavage failure and morphological dysfunction of the cleaved embryos [138]. A study demonstrates the presence of cell membrane-bound GHR on the human oocytes collected from patients undergoing IVF. It has been concluded that GH plays a direct role in improving the quality of oocytes through the upregulation of their own receptors and enhancement of mitochondrial activity. These results support the use of exogenous GH supplementation for the clinical management of patients with a poor ovarian response (POR) [134]. GH plays a significant role in ovulation by increasing the sensitivity of the gonadotropins at an ovarian level and by reducing the incidence of apoptosis in the preovulatory ovarian follicles [139, 140] (Figure 2).

IGF-1 can stimulate proliferation and differentiation of granulosa cells and theca cells as well [141, 142]. IGF receptors have been demonstrated to be absolutely needed for FSH-mediated activation of the prosurvival cascade and subsequent granulosa cell differentiation [143, 144]. In animal models, IGF-1 was found to elevate the number of gap junctions between theca-granulosa cells and granulosa cell-oocyte, promote preantral follicular growth [145, 146], and improve follicular and cell survival [147–149] (Figure 2). Of note, the effects of IGF-1 on ovarian actions may be species-specific. Studies suggest that GH and IGF-1 may act partially independently. GHR knockout mice showed delayed follicular maturation, but follicular IGF-1 levels were normal [150, 151], which has indicated that IGF-1 production can be induced by other hormones (such as gonadotropins and E2) [46, 119]. Female patients with Laron syndrome had delayed puberty, but they achieved full sexual development, and their fertility was not affected [13]. The reason may be the synthesis of local IGF-1 controlled by gonadotropins and E2 which acts in paracrine and autocrine ways. Some investigators conclude that GH affects follicular development through IGF-1 [152]. Although IGF-1 may play a more direct role than GH in the early process of follicular development, the conclusion is not comprehensive enough since GH can modify gene expression and regulate cell metabolism and proliferation independently [153, 154]. It cannot be conclusively assigned which hormone plays a more important role.

The effectiveness of GH on pregnancy outcomes of women who underwent IVF is significant, especially in those who were poor responders under ovarian stimulation [155, 156]. In a study, the number of mature follicles acquired from patients who were tolerant to gonadotropin therapy increased after cotreatment of GH and gonadotropins, suggesting that the ovarian response of those patients enhanced [157]. In another study, patients who received combined therapy of GH and gonadotropins needed lower doses and shorter durations to achieve the same efficacy of ovulation induction than those who were treated with gonadotropins and a placebo [158]. The majority of AGHD women, but not all, require assisted reproductive technologies to induce ovulation. The facilitatory role of GH in the process is emphasized [159]. Nevertheless, it is pointed out that the ovary can sometimes bypass this deficit and that ovulation may occur without GH, suggesting that GH is not the fundamental factor to influence ovulation [159]. Indeed, considering the small sample size, the necessity of GH treatment for infertile women is not clear. It is suggested that infertile women with GHD should accept GH supplementation [160], but data on the outcomes of fertility are scarce.

Among women with polycystic ovary syndrome (PCOS), GH also has an obvious regulatory effect on ovarian function. PCOS is a common endocrine disease, in which hyperandrogenemia is the main factor leading to anovulation. Gonadotropin secretion disorder and insulin resistance lead to a high proportion of infertility. Hyperandrogenemia caused by PCOS may contribute to reduced GH secretion [161]. For infertile PCOS patients with POR to exogenous gonadotropins, GH therapy may enhance their gonadal activity by increasing the activity of IGF-1 [162]. It has been found that the potential mechanism may be that GH administration can increase women's responsiveness to human menopausal gonadotrophin (hMG), who are diagnosed with PCOS [163, 164].

The ovarian cycle and hormones produced by the ovary have a direct and indirect influence on hypothalamic-pituitary actions and endometrial and myometrial changes during implantation and pregnancy [165]. Undoubtedly, the hypothalamic-pituitary-ovarian-endometrial axis is extremely important to the reproductive function of females. The endometrium also responds to GH, with the expression of GHR observed in the endometrial tissue of humans (Figure 2). GHR is expressed in glandular cells of the human endometrium and decidua during the mid and late luteal phases, and GH exerts an important effect on blastocyst implantation [166].

GH has a positive effect on endometrial receptivity (Figure 2). Endometrial receptivity refers to the ability of uterine tissue to allow embryo attachment and implantation, which is closely related to endometrial thickness (EMT) and uterine perfusion [167]. Increasing EMT and promoting uterine perfusion are the key points of endometrial receptivity improvement [168], and thus, high success rates of implantation and pregnancy can be achieved [169]. A recent study showed that GH/IGF-1 may display effects on human endometrial cells to improve proliferation and induce the expression of genes associated with endometrial receptivity such as vascular endothelial growth factor (VEGF) which promotes vascularization [170]. IGF-1 is present in the human reproductive tract and exerts important effects on cellular proliferation and preimplantation development of the human embryo [171]. VEGF is considered one of the most important estrogen-responsive angiogenic factors [172], and it has been demonstrated to act in an autocrine way on endometrial epithelial cell adhesion during implantation [173]. The improvement of endometrial receptivity is also an indirect benefit of GH through the gonadal system, since GH and IGF-1 can increase the levels of estrogens (Figure 2). A prospective study of females (aged <38 years) who underwent frozen-thawed embryo transfer (FET) demonstrated an increase in EMT and serum levels of VEGF as well as an improvement in the clinical pregnancy rate, embryo implantation rate, and live birth rate in the group utilizing additional GH treatment [174]. However, in another study on infertile women with poor endometrial development (EMT <7 mm), while EMT improved, there were no notable changes in pregnancy outcomes [175].

Concerning the gonadal function of women with excess GH, a study on acromegalic women without menopause showed that the estimated duration of acromegaly was longer in patients who had gonadal dysfunction without central hypogonadism than that in acromegalic patients who had normal gonadal function. The levels of anti-Müllerian hormone were low in 64% of acromegalic women of reproductive age, suggesting that excess GH might result in diminished ovarian reserve [176]. However, pregnancy in women with controlled acromegaly can be well achieved, and active acromegaly may not have adverse impacts on the outcomes of pregnancy [177].

#### 2.3.2. Sexual Function of Females

Taking the expression of eNOS in the clitoris and its significance during clitoris erection into account [178], it is reasonable to speculate that GH exerts peripheral impacts as a mediator which promotes the synthesis and action of eNOS, so the production of NO increases. Hence, it promotes vascular smooth muscle relaxation which is responsible for the engorgement of clitoris tissue so that satisfactory sexual performance can be achieved [179].

In relation to the quality of life (QoL), an AGHD-specific questionnaire has been used to evaluate at baseline and after 1 and 2 years of GH therapy in women with severe GHD. The result showed that GH administration was able to particularly improve the ability of sexual arousal [180]. Furthermore, in a recent prospective study, female sexual function was assessed by the Female Sexual Function Index (FSFI, a simple multidimensional scale including 19 questions, divided into 6 areas: desire, arousal, lubrication, orgasm, satisfaction, and pain) [181]. Higher scores indicated better sexual function. The results showed that there was an extremely high prevalence of female sexual dysfunction (FSD) (89%) in 28 AGHD female patients and that FSD was prevalent in all untreated patients; however, rhGH therapy was related to a lower prevalence (77%). The study also found that the overall score of FSFI was significantly higher in treated female patients than that in untreated ones; especially, the desire, arousal, lubrication, and orgasm domain scores were significantly higher in female patients treated with rhGH than those in untreated patients [96]. These findings demonstrate that the sexual function of females with AGHD who are treated with rhGH can be ameliorated. However, considering the estrogen levels of patients [182], the age of patients, and the small-size sample, the positive effect of rhGH on sexual function in AGHD females may be less emphasized than that in AGHD males.

The effects of excessive GH on female sexual function should be considered as well. In a study, 68% of acromegalic patients had FSD by FSFI, while the prevalence of FSD was 28% in healthy women. The total FSFI score and the scores of desire, arousal, orgasm, and satisfaction domains were lower in subjects with acromegaly than those in healthy females. Nevertheless, for women with excess GH, the total FSFI scores and domain scores of patients in the controlled group were not markedly different from those in the uncontrolled group [183]. It is illustrated that the impairment of sexual function induced by excessive GH may be irreversible, even if the levels of GH and IGF-1 are within the normal range for acromegalic patients in remission. Another possible reason is that some complications of acromegaly which may have negative effects on female sexual function are not fully recovered though acromegaly has been controlled through the treatment [184]. Like males, GH can influence female sexual function indirectly. Reduced strength, depression, and fatigue in AGHD can contribute to FSD [113]. Changes in appearances and figures induced by acromegaly play important roles in FSD [114]. Of note, studies about GH effects on female sexual function are limited, since FSD is largely neglected [185], and more confounding factors should be considered. FSD should be taken seriously, and further investigations are needed.

As mentioned above, GH and IGF-1 have many beneficial effects on adult human gonads and the HPG axis; however, they play pathogenetic roles as well. IGF-1 may be related to prostate cancer development and progression [186]. A 2001 study analyzed 48 human breast carcinomas and found that the levels of GHR in neoplastic tissues were higher than those in normal breast tissues [187]. The mitogenic effect of GH is correlated with uterine cancer and cervical cancer. Autocrine GH may be particularly mitogenic as the upregulation of uterine GH in endometriosis and endometrial adenocarcinoma [188]. The degree of autocrine GH expression in endometrial cancer is closely related to tumor invasiveness and metastasis in patients with endometrial cancer [189]. A study that involved 33 young patients (<40 years) with cervical neoplasms has found that more than half of tumor cells express nuclear GHR in 61% of those patients and has suggested that a high degree of nuclear GHR may be a promoter of stimulating the progression of cervical carcinoma cells [190]. Conversely, a review has summarized that papers published so far show that GH treatment is indicated in those patients who have GHD and that it is safe in terms of tumor recurrence [191]. In conclusion, the use of GH should be controlled and the choice of whether to start rhGH treatment or not should be determined according to the characteristics of the patient.

## 3. Conclusions

GH plays important roles in all ages, and IGF-1 is the indispensable mediator. This review summarizes the effects of GH on adult human gonads from two aspects, including reproduction and sexual function. For males, GH has significant effects on augmenting the sensitivity of gonadotropins and improving testicular steroidogenesis. The GH effect on spermatogenesis is less clear, and more explorations are needed. The positive influence of GH on erectile function and sexual function of men who are diagnosed with AGHD is obvious. Excessive GH may do harm to T production and spermatogenesis. Sexual dysfunction is frequent in acromegalic patients. For females, GH can modulate ovarian steroidogenesis and ovarian angiogenesis, enhance ovarian function, and improve endometrial receptivity. GH also contributes to strengthening the sexual function of women with AGHD. Acromegaly may lead to decreased ovarian reserve but seems to have few effects on the outcomes of pregnancy. The prevalence of FSD is high in acromegalic women.

Notably, GH may play a pathological role in the body system since there is evidence that GH causes oncogenic transformation and neoplastic progression. Although adverse reactions are rare, the long-term safety of GH for cancer risks, metabolic disorders, and other unforeseen adverse events should be continuously monitored.

Further studies about the effects of GH on adult human gonads that exclude more confounding factors are needed, which may contribute to detecting AGHD and/or HH earlier, making the relationship between AGHD and HH clearer, and clarifying the indications and safety of rhGH treatment for patients with AGHD and/or HH.
